# Comparing the effectiveness and safety of dual antiplatelet with ticagrelor or clopidogrel in elderly Asian patients with acute myocardial infraction

**DOI:** 10.3389/fcvm.2023.1143509

**Published:** 2023-03-16

**Authors:** Jong-Shiuan Yeh, Wan-Ting Chen, Brian Tomlinson, Weng-Chio Tam, Li-Nien Chien

**Affiliations:** ^1^ Division of Cardiovascular Medicine, Department of Internal Medicine, Taipei Municipal Wan-Fang Hospital, Taipei, Taiwan; ^2^ Division of Cardiology, Department of Internal Medicine, School of Medicine, College of Medicine, Taipei Medical University, Taipei, Taiwan; ^3^ Health Data Analytics and Statistics Center, Office of Data Science, Taipei Medical University, Taipei, Taiwan; ^4^ Faculty of Medicine, Macau University of Science and Technology, Macao SAR, China; ^5^ Department of Cardiology, Centro Hospitalar Conde São Januário, Macao SAR, China; ^6^ Institute of Health and Welfare Policy, College of Medicine, National Yang Ming Chiao Tung University, Taipei, Taiwan; ^7^ Graduate Institute of Data Science, College of Management, Taipei Medical University, Taipei, Taiwan

**Keywords:** acute myocardial infarction, clopidogrel, elder, net adverse clinical events, percutaneous coronary intervention, ticagrelor

## Abstract

**Background:**

Current guidelines recommend potent P2Y12 inhibitors for patients after acute coronary syndrome. However, the data on the efficacy and safety of potent P2Y12 inhibitors in elderly Asian populations was limited. We aimed to investigate the major adverse cardiovascular events (MACE), bleeding events, and net adverse clinical events (NACE) with ticagrelor and clopidogrel in Taiwanese patients aged 65 and older after acute myocardial infarction (AMI).

**Methods:**

This retrospective population-based cohort study was conducted using data from the National Health Insurance Research Database. The AMI patients aged ≥65 years who underwent percutaneous coronary intervention (PCI) and survived after 1 month were included. The patients were separated into 2 cohorts depending on the type of dual antiplatelet therapy (DAPT) they received: ticagrelor plus aspirin (T + A) or clopidogrel plus aspirin (C + A). We used inverse probability of treatment weighting to balance the difference between these 2 study groups. The outcome included all-cause mortality, MACE (cardiovascular death, nonfatal ischemic stroke, and nonfatal myocardial infarction), intracerebral hemorrhage, major bleeding, and NACE which is composed of cardiovascular death, ischemic and hemorrhagic events. The follow-up period was up to 12 months.

**Results:**

From 2013 to 2017, a total of 14,715 patients who met the eligibility criteria were separated into 2 groups: 5,051 for T + A and 9,664 for C + A. Compared to patients with C + A, patients who received T + A had a lower risk of cardiovascular death and all-cause death, with an adjusted HR of 0.57 [95% confidence interval (CI), 0.38–0.85, *p* = 0.006] and 0.58 (95% CI 0.45–0.74, *p* < 0.001), respectively. No differences were found in MACE, intracranial and major bleeding between the 2 groups. In addition, the patients with T + A had a lower risk of NACE with an adjusted HR of 0.86 (95% CI 0.74–1.00, *p* = 0.045)

**Conclusion:**

Among elderly AMI patients receiving DAPT after successful PCI, ticagrelor was a more favorable P2Y12 inhibitor than clopidogrel because of lowering the risk of death and NACE without increasing the risk of severe bleeding. Ticagrelor is an effective and safe P2Y12 inhibitor in Asian elderly survivors after PCI.

## Introduction

The Asia-Pacific region is populated by more than 4.2 billion inhabitants, equivalent to 60% of the world's population. Acute coronary syndrome (ACS) is now a major cause of death and disability in this region as the Western conturies (1). Dual antiplatelet therapy (DAPT), consisting of aspirin combined with a P2Y12 inhibitor, has been the standard of care in preventing coronary and cerebrovascular thrombotic events in patients with chronic coronary syndrome and ACS, but choosing the optimal treatment combination and balance between the ischemic and bleeding risk of treatment duration have become difficult challenges (2–4). Current guidelines recommend using risk stratification instruments for tailoring treatment duration and composition (5–9).

In the global randomized controlled Phase III PLATO trial, ticagrelor, a more potent P2Y12 receptor antagonist, has been demonstrated to reduce the composite end-point of myocardial infarction (MI), stroke, and death from cardiovascular causes across different age groups (10). However, potent P2Y12 inhibitor-related bleeding occurred more frequently in elder ACS patients, than clopidogrel-related bleeding in real-world practice (11). And yet, no guideline suggests the choice of DAPT by age group. To fill in the knowledge gap, our study aimed to examine the effectiveness and safety of ticagrelor or clopidogrel combined with aspirin as dual antiplatelet therapy among AMI patients aged 65 years and older who underwent percutaneous coronary intervention (PCI).

## Materials and methods

### Study design and data sources

This retrospective population-based cohort study was conducted by using data from the National Health Insurance Research Database (NHIRD) that was maintained by the Health and Welfare Data Science Center, Ministry of Health and Welfare. The NHIRD was a claims-based database that covered healthcare utilization and costs for 99% of residents in Taiwan. The NHIRD files included inpatient, outpatient, and pharmaceutical claims. The data of disease diagnosis was coded by the International Classification of Diseases, Ninth Revision, Clinical Modification (ICD-9-CM), and the Tenth Version (ICD-10-CM) after 2016. The data of prescription drugs can be classified based on Anatomical Therapeutic Chemical (ATC) classification. In addition, the enrollment files of beneficiaries and providers were included. The data period used in this study was from 2013 to 2017. The study protocol had been reviewed and approved by Taipei Medical University Joint Institutional Review Board (N202205097).

### Study cohort

**Figure 1:**
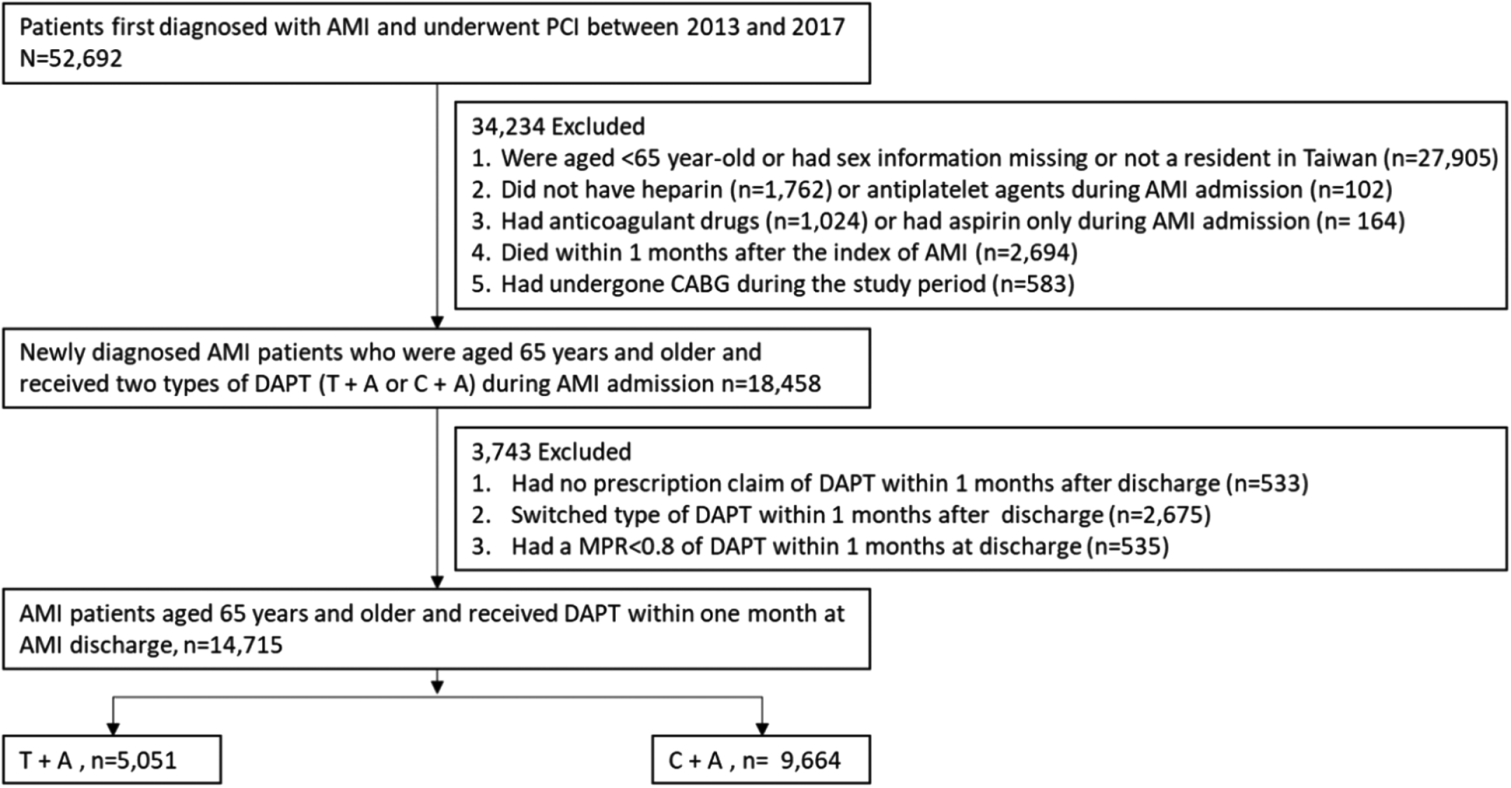
Patient selection process. A, aspirin; AMI, acute myocardial infraction; C, clopidogrel; CABG, coronary artery bypass graft; DAPT, dual antiplatelets therapy; MPR, medication possession ratio; PCI, percutaneous coronary intervention; T, ticagrelor.

Patients first diagnosed with AMI, including ST elevation (STEMI) and non-ST elevation myocardial infarction (NSTEMI), and who underwent PCI between 2013 and 2017 were included. The period was chosen to follow the regulations of the National Health Insurance Administration because ticagrelor was first reimbursed for treating patients with AMI after July 1st, 2013. The reimbursed DAPT duration was 9 months after AMI. Of these patients, we added several exclusion criteria to ensure that the patients were eligible for the current study, including (1) age less than 65 years, sex information missing, or not a resident in Taiwan; (2) did not have heparin or antiplatelet agents during the AMI admission; (3) had anticoagulant drugs or had aspirin only during the AMI admission; (4) died within a month after the index AMI; and (5) had a history of coronary artery bypass graft during the study period. Of the patients, we excluded those who had no prescription claim of DAPT, switched the type of DAPT, or had a medication possession ratio (MPR) <0.8 within a month after AMI discharge. MPR is defined as the total number of days covered by filled prescriptions divided by a predefined period (i.e., 30 days). The value of MPR ≥0.8 suggests a significantly higher adherence (12). Figure 1 depicts the process of patient selection in detail.

### Comorbidities and medications

The disease diagnosis codes for baseline comorbidities and ATC codes for medications were provided in Supplementary Table S1. Baseline cardiovascular and bleeding risks were assessed at the time of inclusion. We considered the risk of hypertension, diabetes, hyperlipidemia, heart failure, and prior stroke/transient ischemic stroke. Also, we combined comorbidity information into the CHA_2_DS_2_-VASC score (congestive heart failure, hypertension, age ≥75 years, diabetes, stroke/TIA, vascular disease, age 65–74 years, sex category) for quantifying thromboembolic risk (13). To assess the risk of bleeding, a history of anemia, bleeding, the ORBIT score (age ≥74 years, anemia, bleeding history, GFR <60 ml/min/1.73 m^2^, treatment with antiplatelet therapy), and HAS-BLED score (hypertension, abnormal renal/liver function, stroke, bleeding history or predisposition, labile international normalized ratio, age >65 years, drugs/alcohol concomitantly) were used (14, 15). Both the ORBIT score and HAS-BLED score can predict major bleeding better than other risk scores and were validated in large studies. To control the confounding effect due to the medication history that might associate with cardiovascular outcomes or bleeding events additionally, we considered whether patients had DAPT or NSAIDs more than 3 months within a year before the AMI admission (16, 17).

### Study outcomes

The outcomes of interest included major adverse cardiovascular events (MACE), cardiovascular death, and all-cause death to evaluate the effectiveness of therapy. MACE is defined as a composite of nonfatal ischemic stroke nonfatal MI, and cardiovascular death. The events of intracerebral hemorrhage (ICH) and major bleeding were considered to assess the safety of the treatment. The definition of major bleeding was hospitalized due to bleeding, symptomatic bleeding in a critical area or organ, or bleeding leading to transfusion of more than 2 units of packed red blood cells. We also analyzed the net adverse clinical events (NACE), composed of cardiovascular death, ischemic events (recurrent nonfatal MI or nonfatal ischemic stroke), and hemorrhagic events (Intracerebral hemorrhage or major bleeding). The follow-up period was up to 12 months. The disease diagnosis codes for study outcomes are provided in Supplementary Table S1.

### Statistical analysis

Baseline characteristics were analyzed using standardized mean difference. A standardized mean difference of greater than 0.1 indicates the non-negligible difference between the two groups. Inverse probability of treatment weighted (IPTW) analysis was conducted to adjust for differences in baseline characteristics between the two cohorts. Cox proportional hazard regression adjusted for baseline covariates and medications listed in Table 1 were used to estimate the hazard ratio (HR) between two treatment regimens. Each patient was followed from the date of AMI discharge to the date of the event of interest or up to 1 year of follow-up. All the variables were included in multivariable Cox analyses. All analyses were performed using SAS/STAT 9.4 (SAS Institute Inc., Cary, NC) and STATA 14 (Stata Corp LP, College Station, TX). A *p* value <0.05 was considered significant.

**Table 1:** Baseline characteristics of the elderly AMI patients who received aspirin with ticagrelor or clopidogrel before and after IPTW.

	Before IPTW			After IPTW		
	T + A	C + A		T + A	C + A	
Sample size, *n*	5,051	9,664		5,051	9,664	
	**%**	**%**	**SMD**	**%**	**%**	**SMD**
Age, year (mean, SD)	(73.7, 6.9)	(76.1, 7.5)	0.328	(75.6, 7.3)	(75.3, 7.4)	0.036
65–74	58.5	45.6	0.262	48.3	50.0	0.034
≥75	41.5	54.4	0.262	51.7	50.0	0.034
Male (%)	73.4	65.3	0.176	65.3	67.9	0.055
**Comorbidities (%)**
Hypertension	73.0	76.5	0.079	76.7	75.4	0.032
Diabetes	42.1	45.6	0.072	45.0	44.5	0.010
Hyperlipidemia	52.1	45.2	0.139	47.6	47.4	0.004
Heart failure	19.1	27.1	0.189	26.3	24.5	0.041
Stroke/TIA	8.6	11.5	0.096	11.6	10.6	0.033
COPD	10.4	12.3	0.058	11.1	11.6	0.015
Anemia	4.7	7.1	0.100	7.6	6.3	0.051
History of bleeding	5.1	8.2	0.122	9.0	7.2	0.065
Abnormal renal function	13.3	20.2	0.187	19.0	17.9	0.029
Abnormal liver function	3.8	3.4	0.022	3.3	3.6	0.013
Cancer	6.3	8.0	0.067	7.5	7.4	<0.001
Autoimmune diseases	0.1	0.1	0.005	0.2	0.1	0.018
**Previous medication use (%)**
DAPT	33.0	41.8	0.182	42.1	38.9	0.064
NSAIDs	39.3	38.7	0.013	38.4	39.1	0.014
CHADS_2_-VASc score, (mean, SD)	(4.2, 1.3)	(4.6, 1.4)	0.298	(4.5, 1.4)	(4.5, 1.4)	0.060
ORBIT score, (mean, SD)	(1.1, 1.1)	(1.5, 1.3)	0.326	(1.5, 1.3)	(1.4, 1.3)	0.094
HAS-BLED-score, (mean, SD)	(2.8, 1.2)	(3.1, 1.2)	0.211	(3.1, 1.2)	(3.0, 1.2)	0.065
**Year of AMI diagnosis (%)**
2013	3.2	28.1	0.727	22.3	19.6	0.065
2014	14.5	21.5	0.184	18.9	19.2	0.008
2015	22.6	17.2	0.134	18.6	19.2	0.015
2016	29.6	16.4	0.317	19.9	20.9	0.023
2017	30.1	16.7	0.320	20.3	21.1	0.020

A, aspirin; AMI, acute myocardial infraction; C, clopidogrel; COPD, chronic obstructive pulmonary disease; DAPT, dual antiplatelets therapy; IPTW, inverse probability treatment weighting; NSAIDs, non-Steroidal anti-inflammatory drugs; SD, standard deviation; SMD, standardized mean difference; T, ticagrelor; TIA, transient ischemic attack.

## Results

Among 52,692 patients first diagnosed with AMI and underwent PCI between 2013 and 2017, a total of 14,715 patients, including STEMI (49.7%) and NSTEMI (50.3%) met the eligibility criteria. The patients were separated into two cohorts depending on the type of DAPT: 5,051 for ticagrelor plus aspirin (T + A) and 9,664 for clopidogrel plus aspirin (C + A) (Figure 1).

Baseline characteristics are shown in Table 1. Before IPTW, patients who received T + A were younger and had a higher percentage of males but a lower percentage of comorbidities, CHA_2_DS_2_-VASc, ORBIT, and HAS-BLED scores. Also, more patients had received T + A in more recent years. The mean (±standard deviation) DAPT duration of T + A and C + A was 7.1 (±3.9) and 7.9 (±3.7) months (*p* = 0.218), respectively. After IPTW, baseline characteristics were well-balanced between the 2 groups.

**Figure 2:**
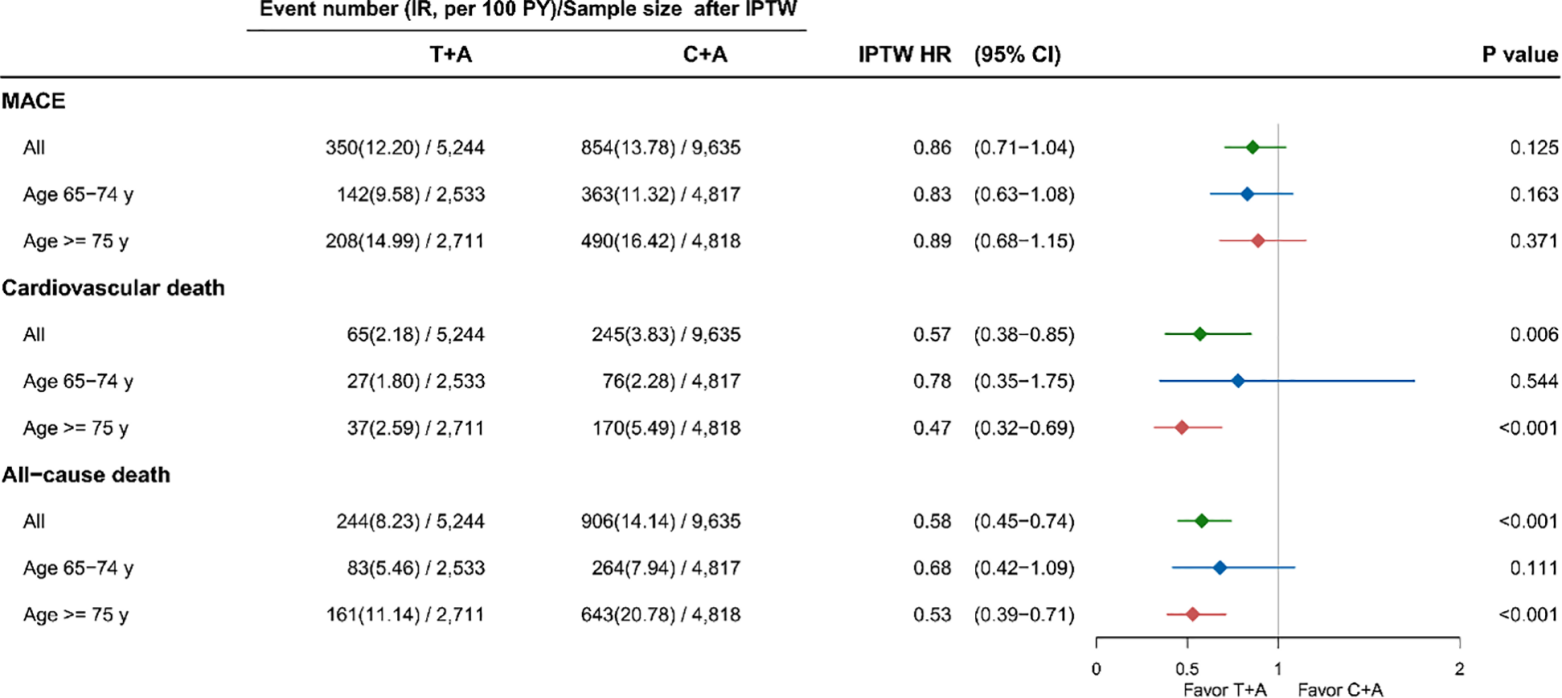
Effectiveness of ticagrelor and clopidogrel on MACE, cardiovascular death and all-cause death among AMI patients. A, aspirin; AMI, acute myocardial infraction; C, clopidogrel; HR, hazard ratio; IPTW, inverse probability treatment weighting; MACE, major adverse cardiovascular events; T, ticagrelor; y, years.

The incidence and relative risks of MACE, cardiovascular death, and all-cause death are shown in Figure 2. Compared to patients with C + A, patients with T + A had a lower risk of cardiovascular death and all-cause death, with an adjusted HR of 0.57 (95% confidence interval 0.38–0.85, *p* = 0.006) and 0.58 (95% confidence interval 0.45–0.74, *p* < 0.001). The MACE events rate was lower in the T + A group, although the *p*-value was insignificant. The recurrent MI and ischemic stroke events rate were comparable in 2 groups (Supplementary Figure S1). After stratifying the analysis into different age groups, the protective effect on cardiovascular death and all-cause death was observed in patients aged 75 years and over in the T + A group.

**Figure 3:**
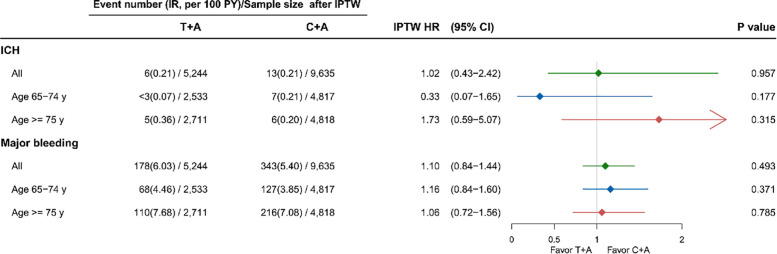
Safety of ticagrelor and clopidogrel on ICH and major bleeding among AMI patients. A, aspirin; AMI, acute myocardial infraction; C, clopidogrel; HR, hazard ratio; ICH, intracerebral hemorrhage; IPTW, inverse probability treatment weighting; T, ticagrelor; y, years.

**Figure 4:**
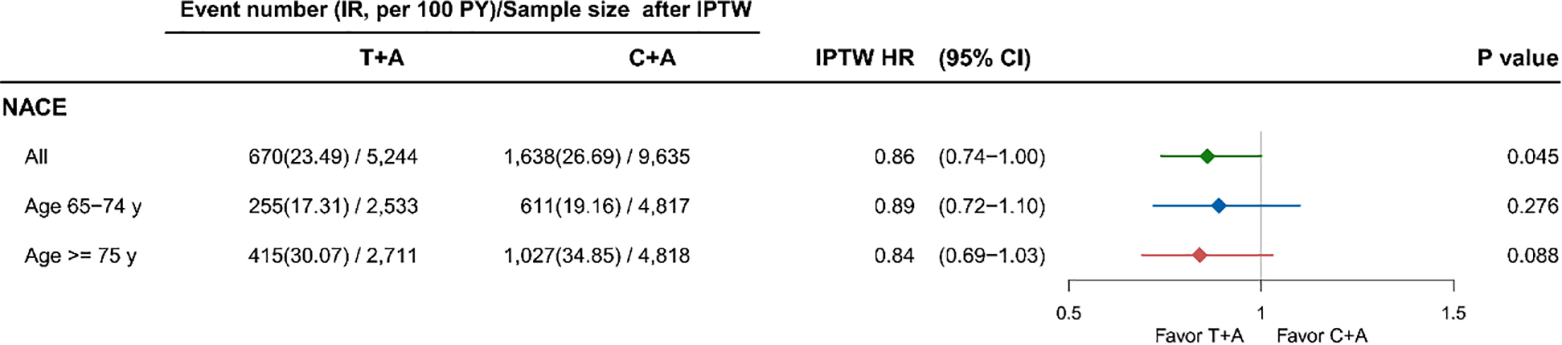
Outcome of ticagrelor and clopidogrel on NACE among AMI patients. A, aspirin; AMI, acute myocardial infraction; C, clopidogrel; HR, hazard ratio; ICH, intracerebral hemorrhage; IPTW, inverse probability treatment weighting; NACE, net adverse clinical event; STEMI, ST segment elevation myocardial infarction; T, ticagrelor; y, years.

The incidence and relative risks of ICH and major bleeding are shown in Figure 3. No differences were found in ICH and major bleeding between the 2 groups. The results were also similar in both age groups. The Figure 4 revealed the patient with T + A had lower risk of NACE, with an adjusted HR of 0.86 (95% confidence interval 0.74–1.00, *p* = 0.045) while no different in age groups.

## Discussion

This nationwide population-based cohort study found that ticagrelor was superior to clopidogrel in lowering the risk of death and NACE while not increasing the risk of marjor bleeding among elderly AMI patients after PCI. The phenomenon was found in those aged 65 years and older.

Studies have shown that compared to clopidogrel, the more potent P2Y12 inhibitors, including prasugrel and ticagrelor, further reduced the risk of death, MI, and stroke in ACS patients (18, 19). But, an increase in non-critical major bleeding risk was found in the patients with these more potent P2Y12 inhibitors, which might indicate a trade-off between the anti-thrombotic benefit and the bleeding side effects (20). The hemorrhagic risk rises exponentially from the seventh decade, leading to prolonged hospital stay, increased medical expenses, and disproportionate risk of death (21). A previous Taiwanese retrospective national cohort study revealed a similar cardiovascular benefit of ticagrelor while limited to NSTEMI patients (22). In our study, we included both STEMI and NSTEMI patients and focused our study populations on the elderly because of both high ischemic and bleeding risk in this vulnerable population (23). Furthermore, we analyzed the risk of NACE to weigh the gains and losses of different DAPT strategy. Fortunately, the current large-scale study found the effectiveness and safety of ticagrelor were comparable with clopidogrel in elderly AMI patients based on real-world data.

The fear of increased bleeding risk and the intrinsic frailty of this aging population often lead physicians to prescribe a less potent P2Y12 inhibitor. As a result, those patients might be potentially undertreated and exposed to a higher incidence of thrombotic complications (24–26). According to our data, the percentage of ticagrelor plus aspirin as a DAPT regimen was lower in those aged 75 and older, which was one-third of elderly AMI patients; however, the trend has been increasing gradually recently. One of the possible reasons might be the international and domestic guidelines’ recommendation of ticagrelor over clopdogrel in ACS patients (27–29).

This current study confirmed the significant survival benefit of ticagrelor over clopidogrel and similar major bleeding risk between treatment groups. The similar findings were observed in the PLATO trial although a recent randomized open-label study, POPular-AGE study, including patients aged ≥70 years with non-ST elevation ACS showed similar thrombotic events between ticagrelor and clopidogrel but less bleeding in the clopidogrel group (11). The possible reason for the discrepancy might be due to the less adherence to ticagrelor. A recent analysis from the PARIS (Patterns of Non-Adherence to Anti-Platelet Regimens in Stented Patients) registry suggested that those patients who stop DAPT therapy due to “disruption” (nonadherence or bleeding), have the highest risk of major cardiovascular events (30). In the POPular-AGE trials, the adherence to ticagrelor was lower than that to clopidogrel, with only 53% of patients continuing the more potent P2Y12 inhibitor compared with 78% for clopidogrel during the 12-month follow-up. We only included the patients with a medication possession ratio (MPR) ≥ 0.8 for both DAPT treatment groups; therefore, the benefit of ticagrelor in preventing major cardiovascular events might not be neutralized by the early disruption of this potent P2Y12 inhibitor.

Meanwhile, ticagrelor might have pleiotropic effects that go beyond antiplatelet action. A recent study has indicated that ticagrelor may have antimicrobial activity in a mouse model (31). But, only a few clinical studies have been focused on the potential bactericidal effect (32, 33). We additionally examined the risk of infection events, including pneumonia, urinary tract infection (UTI), and cellulitis after AMI discharge. A decreased risk of pneumonia hospitalization was observed in patients with T + A (Supplementary Figure S2) Compared to patients with C + A, those with T + A had a lower risk of pneumonia (adjusted HR of 0.7, 95% confidence interval 0.56–0.86, *p* = 0.001). These pleomorphic effects of ticagrelor beyond what we know about the platelet inhibition might provide some hypothesis for our study's survival benefit of ticagrelor over clopidogrel (34, 35). Further studies should be launched and investigated the anti-infective or anti-inflammatory activity of ticagrelor.

Although short duration or de-escalated DAPT strategy might reduce bleeding without increasing cardiovascular events in some studies, but current guideline still recommend DAPT consisting of a potent P2Y12 receptor inhibitor in addition to aspirin for 12 months in acute coronary syndrome patients after PCI, irrespective of the stent type, unless there are contraindications adequate (36–38). Studies are deserved to evaluate the effiicacy and safety of short duration DAPT in elder population.

There were some limitations in this study. First, the NHIRD does not have certain patient information, such as risk behaviors, diet, and physical activities, which might be associated with the outcomes of interest. Second, the NHIRD does not contain clinical information, such as angiographic findings during PCI, the extent of coronary artery disease, and the severity of AMI at admission. Consequently, we could not adjust for the severity of AMI nor could we know whether the AMI admission was planned or not, which might induce non-differential misclassification bias. We did not follow BARC or GUSTO, or TIMI criteria for bleeding classification because of the limitation of NHIRD that laboratory data like the hemoglobin or other exact clinical records was unable to obtain. Therefore, some outcome measurement like minor bleeding might be underestimated. In this study, we defined the major bleeding as being hospitalized due to bleeding, bleeding in a critical organ, or bleeding leading to transfusion that were comparable to the major bleeding of TIMI or BARC 2–3 bleeding criteria. The similar method has been used in the claim-based studies (20, 39). Third, only observed variables were considered in the IPTW; therefore, hidden bias was possible. Finally, we only included Taiwanese patients and we did not include patients with known coronary artery disease or acute coronary syndrome history because they are usually more complicated in clinical condition that may interfere the physicians’ decision on DAPT stategies. Therefore, the results might not be generalizable to other patient populations.

## Conclusion

Using the nationwide cohort study, we found that in Asian patients aged 65 years or older presenting with AMI, ticagrelor provided a more favorable outcome than clopidogrel, because it was associated with a lower cumulative rate of all-cause mortality, cardiovascular death and NACE without an increase of intracranial or major bleeding.

## Data Availability

The raw data supporting the conclusions of this article will be made available by the authors, without undue reservation.

## Supplementary material

**Supplement Figure 1** Effectiveness of ticagrelor and clopidogrel on MI and ischemic stroke among AMI patients Abbreviation: A=aspirin, AMI=acute myocardial infraction, C=clopidogrel, HR=hazard ratio, IPTW=inverse probability treatment weighting, MI=myocardial infarction, T=ticagrelor, y=years.

**Supplementary Figure 2** Effectiveness of ticagrelor and clopidogrel on pneumonia, UTI and Cellulitis among AMI patients after the AMI discharge Abbreviation: A=aspirin, AMI=acute myocardial infraction, C=clopidogrel, HR=hazard ratio, IPTW=inverse probability treatment weighting, T=ticagrelor, UTI= urinary tract infection, y=years.
