# Supplementary figures and images for: Comparing the effectiveness and safety of dual antiplatelet with ticagrelor or clopidogrel in elderly Asian patients with acute myocardial infraction
Source: Front Cardiovasc Med. 2023 Mar 16;10:1143509. doi: 10.3389/fcvm.2023.1143509 (PMC10060791; doi:10.3389/fcvm.2023.1143509)

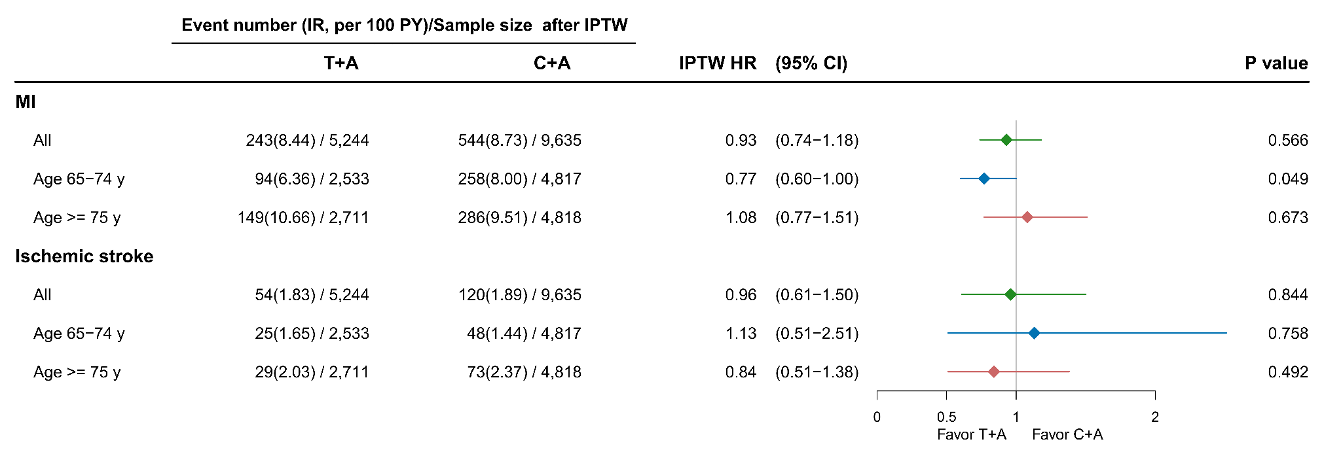

Supplement: Supplementary file 1 [file Image1.tiff]

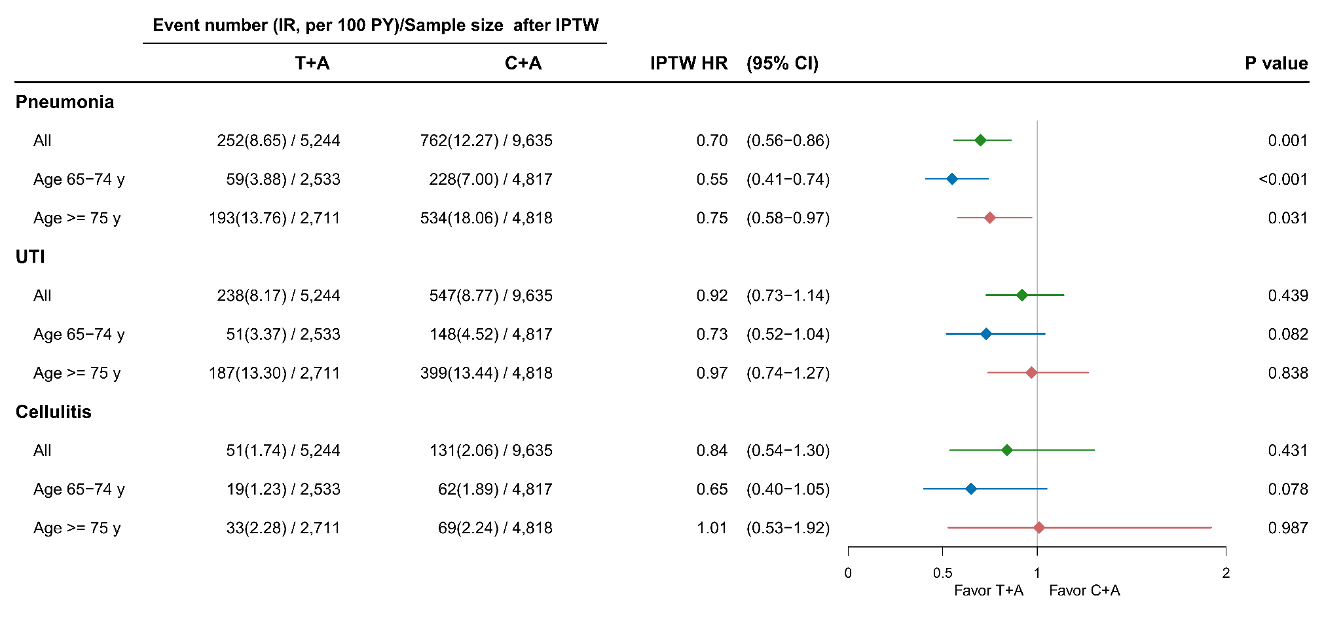

Supplement: Supplementary file 2 [file Image2.tiff]
