# Supplementary material for: Comparing the effectiveness and safety of dual antiplatelet with ticagrelor or clopidogrel in elderly Asian patients with acute myocardial infraction
Source: Front Cardiovasc Med. 2023 Mar 16;10:1143509. doi: 10.3389/fcvm.2023.1143509 (PMC10060791; doi:10.3389/fcvm.2023.1143509)
Supplement: Supplementary file 3 [file Table1.pdf]

Table S1. ICD-9-CM and ICD-10-CM codes for disease diagnostic coding and ATC codes for medication use		
**Comorbidities**	ICD-9-CM	ICD-10-CM
Hypertension	401-405	I10, I110, I119, I120, I129, I130, I1310, I1311, I132, I150, I151, I152, I158, I159, N262
Diabetes	250	E0800, E0801, E08311, E08319, E08321, E08329, E08331, E08339, E08341, E08349, E08351, E08359, E0836, E0839, E0840, E0841, E0842, E0843, E0844, E0849, E0851, E0852, E0859, E08641, E088, E0900, E0901, E09311, E09319, E09321, E09329, E09331, E09339, E09341, E09349, E09351, E09359, E0936, E0939, E0940, E0941, E0942, E0943, E0944, E0949, E0951, E0952, E0959, E09641, E098, E1010, E1011, E1021, E1022, E1029, E10311, E10319, E1036, E1039, E1040, E1041, E1044, E1049, E1051, E1052, E1059, E10610, E10618, E10620, E10621, E10622, E10628, E10630, E10638, E10641, E10649, E1065, E1069, E108, E109, E1100, E1101, E1121, E1122, E1129, E11311, E11319, E11321, E11329, E11331, E11339, E11341, E11349, E11351, E11359, E1136, E1139, E1140, E1141, E1142, E1143, E1144, E1149, E1151, E1152, E1159, E11610, E11618, E11620, E11621, E11622, E11628, E11630, E11638, E11641, E11649, E1165, E1169, E118, E119, E1300, E1301, E1311, E1321, E1322, E1329, E13311, E13319, E13321, E13329, E13331, E13339, E13341, E13349, E13351, E13359, E1336, E1339, E1340, E1341, E1342, E1343, E1344, E1349, E1351, E1352, E1359, E13641, E138, E139
Hyperlipidemia	272.0 – 272.4	E780, E781, E782, E783, E784, E785
Heart failure	428	I501, I5020, I5021, I5022, I5023, I5030, I5031, I5032, I5033, I5040, I5041, I5042, I5043, I509
COPD	490 – 492, 494, 496	J40, J410, J411, J418, J42, J430, J431, J432, J438, J439, J440, J441, J449, J470, J471, J479
Anemia	280, 281, 285.2, 285.8, 285.9	D500, D501, D508, D509, D510, D511, D512, D513, D518, D519, D520, D521,

		D528, D529, D530, D531, D532, D538, D539, D630, D631, D638, D644, D6489, D649
History of bleeding	456.0, 456.20, 530.7, 530.82, 531.0, 531.2, 531.4, 531.6, 532.0, 532.2, 532.4, 532.6, 533.0, 533.2, 533.4, 533.6, 534.0, 534.2, 534.4, 534.6, 535.01, 535.11, 535.21, 535.31, 535.41, 535.51, 535.61, 537.83, 562.02, 562.03, 562.12, 562.13, 569.3, 569.85, 578.0, 578.1, 578.9, 430, 431, 432, 432.0, 432.1, 432.9, 767.0, 852–852.5, 853–853.1	I8501, I8511, K226, K228, K250, K252, K254, K256, K260, K262, K264, K266, K270, K272, K274, K276, K280, K282, K284, K286, K2901, K2921, K2931, K2941, K2951, K2961, K2971, K2981, K2991, K31811, K3182, K5281, K5521, K5660, K5701, K5711, K5713, K5721, K5731, K5733, K5781, K5791, K5793, K625, K920, K921, K922, I6000, I6001, I6002, I6010, I6011, I6012, I6020, I6021, I6022, I6030, I6031, I6032, I604, I6050, I6051, I6052, I606, I607, I608, I609, I610, I611, I612, I613, I614, I615, I616, I618, I619, I6200, I6201, I6202, I6203, I621, I629, P100, P101, P104, P108, P109, P110, P112, P119, P524, P526, P528, P529, S0190XA, S06340A, S06341A, S06342A, S06343A, S06344A, S06345A, S06346A, S06347A, S06348A, S06349A, S06350A, S06351A, S06352A, S06353A, S06354A, S06355A, S06356A, S06357A, S06358A, S06359A, S06360A, S06361A, S06362A, S06363A, S06364A, S06365A, S06366A, S06367A, S06368A, S06369A, S064X0A, S064X1A, S064X2A, S064X3A, S064X4A, S064X5A, S064X6A, S064X7A, S064X8A, S064X9A, S065X0A, S065X1A, S065X2A, S065X3A, S065X4A, S065X5A, S065X6A, S065X7A, S065X8A, S065X9A, S066X0A, S066X1A, S066X2A, S066X3A, S066X4A, S066X5A, S066X6A, S066X7A, S066X8A, S066X9A
Abnormal renal function	582, 583-583.7, 585, 586, 588	N030, N031, N032, N033, N034, N035, N036, N037, N038, N039, N052, N053, N054, N055, N059, N062, N063, N064, N065, N072, N073, N074, N075, N08, N171, N172, N184, N185, N186, N189, N19, N250, N251, N2581, N2589, N259
Abnormal liver function	571.2, 571.4-571.6	K702, K7030, K7031, K730, K731, K732, K738, K739, K740, K741, K742, K743, K744, K745, K7460, K7469, K754
Cancer	140-172, 174.x-195.8, 200-208	C00-C75, D00-D49
Autoimmune	279.4	D8982, D8989

diseases		
**Medication use**		
DAPT	B01AC	
NSAIDs	M01AA, M01AB, M01AC, M01AE, M01AG, M01AH, M01AX	
**Outcomes**		
myocardial infraction	410, 414.00, 414.01, 414.02, 414.03, 414.04, 414.05	I21, I22, I25
Ischemic stroke	433, 434, 436	I6300, I63011, I63012, I63019, I6302, I63031, I63032, I63039, I6309, I6310, I63111, I63112, I63119, I6312, I63131, I63132, I63139, I6319, I6320, I63211, I63212, I63219, I6322, I63231, I63232, I63239, I6329, I6330, I63311, I63312, I63319, I63321, I63322, I63329, I63331, I63332, I63339, I63341, I63342, I63349, I6339, I6340, I63411, I63412, I63419, I63421, I63422, I63429, I63431, I63432, I63439, I63441, I63442, I63449, I6349, I6350, I63511, I63512, I63519, I63521, I63522, I63529, I63531, I63532, I63539, I63541, I63542, I63549, I6359, I636, I638, I639, I6501, I6502, I6503, I6509, I651, I6521, I6522, I6523, I6529, I658, I659, I6601, I6602, I6603, I6609, I6611, I6612, I6613, I6619, I6621, I6622, I6623, I6629, I663, I668, I669, I6789
Major bleeding		
ICH or hemorrhagic stroke	430, 431, 432	I6000, I6001, I6002, I6010, I6011, I6012, I6020, I6021, I6022, I6030, I6031, I6032, I604, I6050, I6051, I6052, I606, I607, I608, I609, I610, I611, I612, I613, I614, I615, I616, I618, I619, I6200, I6201, I6202, I6203, I621, I629
Gastrointestinal bleeding	456.0, 456.20, 530.7, 530.82, 531.0, 531.2, 531.4, 531.6, 532.0, 532.2, 532.4, 532.6, 533.0, 533.2, 533.4, 533.6, 534.0, 534.2,	I8501, I8511, K226, K228, K250, K252, K254, K256, K260, K262, K264, K266, K270, K272, K274, K276, K280, K282, K284, K286, K2901, K2921, K2931, K2941, K2951, K2961, K2971, K2981, K2991, K31811, K3182, K5281, K5521, K5660, K5701, K5711, K5713, K5721, K5731, K5733, K5781, K5791, K5793, K625, K920, K921, K922

	534.4, 534.6, 535.01, 535.11, 535.21, 535.31, 535.41, 535.51, 535.61, 537.83, 562.02, 562.03, 562.12, 562.13, 569.3, 569.85, 578.0, 578.1, 578.9	
Other non-critical site bleeding	287.8, 287.9, 599.7, 596.7, 770.3, 784.7, 784.8, 786.3	D698, D699, N3289, P260, P261, P268, P269, R040, R041, R042, R0481, R0489, R049, R310, R311, R312, R319
Intracranial bleeding	430, 431, 432, 432.0, 432.1, 432.9, 767.0, 852– 852.5, 853–853.1	I6000, I6001, I6002, I6010, I6011, I6012, I6020, I6021, I6022, I6030, I6031, I6032, I604, I6050, I6051, I6052, I606, I607, I608, I609, I610, I611, I612, I613, I614, I615, I616, I618, I619, I6200, I6201, I6202, I6203, I621, I629, P100, P101, P104, P108, P109, P110, P112, P119, P524, P526, P528, P529, S0190XA, S06340A, S06341A, S06342A, S06343A, S06344A, S06345A, S06346A, S06347A, S06348A, S06349A, S06350A, S06351A, S06352A, S06353A, S06354A, S06355A, S06356A, S06357A, S06358A, S06359A, S06360A, S06361A, S06362A, S06363A, S06364A, S06365A, S06366A, S06367A, S06368A, S06369A, S064X0A, S064X1A, S064X2A, S064X3A, S064X4A, S064X5A, S064X6A, S064X7A, S064X8A, S064X9A, S065X0A, S065X1A, S065X2A, S065X3A, S065X4A, S065X5A, S065X6A, S065X7A, S065X8A, S065X9A, S066X0A, S066X1A, S066X2A, S066X3A, S066X4A, S066X5A, S066X6A, S066X7A, S066X8A, S066X9A
Other critical site bleeding	362.81, 363.61, 363.62, 376.32, 379.23, 423.0, 459.0, 568.81, 719.1	H05231, H05232, H05233, H05239, H31301, H31302, H31303, H31309, H31311, H31312, H31313, H31319, H3560, H3561, H3562, H3563, H4310, H4311, H4312, H4313, I312, K661, M2500, M25011, M25012, M25019, M25021, M25022, M25029, M25031, M25032, M25039, M25041, M25042, M25049, M25051, M25052, M25059, M25061, M25062, M25069, M25071,

		M25072, M25073, M25074, M25075, M25076, M2508, R58
Infections		
Pneumonia	480, 481, 482, 483, 484, 485, 486	A221, A3791, A481, B250, B440, J120, J121, J122, J123, J1281, J1289, J129, J13, J14, J150, J151, J1520, J15211, J1529, J153, J154, J155, J156, J157, J158, J159, J160, J168, J17, J180, J181, J188, J189
UTI	599.0	N390
Cellulitis	682	K122, L0201, L0211, L02211, L02212, L02213, L02214, L02215, L02216, L02219, L0231, L02411, L02412, L02413, L02414, L02415, L02416, L02419, L02511, L02512, L02519, L02611, L02612, L02619, L02811, L02818, L0291, L03111, L03112, L03113, L03114, L03115, L03116, L03119, L03121, L03122, L03123, L03124, L03125, L03126, L03129, L03211, L03212, L03221, L03222, L03311, L03312, L03313, L03314, L03315, L03316, L03317, L03319, L03321, L03322, L03323, L03324, L03325, L03326, L03327, L03329, L03811, L03818, L03891, L03898, L0390, L0391, L983

Abbreviation: ATC= anatomical therapeutic chemical, AMI=acute myocardial infraction, COPD=chronic obstructive pulmonary disease, DAPT=dual antiplatelets therapy, ICD-9-CM=international classification of disease, ninth revision, clinical modification, ICH= intracerebral hemorrhage, UTI=urinary tract infection, NSAIDs= non-Steroidal anti-inflammatory drugs, TIA= transient ischemic attack
